# A Reliability-Based Track Fusion Algorithm

**DOI:** 10.1371/journal.pone.0126227

**Published:** 2015-05-07

**Authors:** Li Xu, Liqiang Pan, Shuilin Jin, Haibo Liu, Guisheng Yin

**Affiliations:** 1 College of Computer Science and Technology, Harbin Engineering University, Harbin, Heilongjiang, China; 2 School of Computer Science and Technology, Harbin Institute of Technology, Harbin, Heilongjiang, China; 3 Department of Mathematics, Harbin Institute of Technology, Harbin, Heilongjiang, China; The University of Science and Technology of China, CHINA

## Abstract

The common track fusion algorithms in multi-sensor systems have some defects, such as serious imbalances between accuracy and computational cost, the same treatment of all the sensor information regardless of their quality, high fusion errors at inflection points. To address these defects, a track fusion algorithm based on the reliability (TFR) is presented in multi-sensor and multi-target environments. To improve the information quality, outliers in the local tracks are eliminated at first. Then the reliability of local tracks is calculated, and the local tracks with high reliability are chosen for the state estimation fusion. In contrast to the existing methods, TFR reduces high fusion errors at the inflection points of system tracks, and obtains a high accuracy with less computational cost. Simulation results verify the effectiveness and the superiority of the algorithm in dense sensor environments.

## Introduction

In the field of track fusion, Measurement Fusion (MF) [1], Simple Fusion (SF) [2]and Weighted Covariance Fusion (WCF) [3] are the most representative track fusion algorithms. MF possesses the advantage of simple idea and small computational cost, but lower accuracy. SF was proposed by Siger and has a notable advantage, high efficiency. However, the accuracy of SF is still not high. WCF was proposed by Bar-Shalom, which is also a classical algorithm. It is characterized by high accuracy, but heavy computational cost. Until now, some performances of them are still the objectives pursued. So, many new algorithms were compared with them on performance. In recent years, many new approaches appeared in the field of track fusion. For examples, Yuan, Dong and Wang gave a fusion algorithm based on an adaptive neuro-fuzzy inference system, which combines the merits of fuzzy logic and neural network [4]. Stienne, Reboul, Azmani, Choquel and Benjelloun assumed the error of angular measurements follow a von Mises distribution and proposed a multi-sensor fusion operator [5]. Duan and Li presented two optimal distributed fusion algorithms by taking linear transformation of the raw sensor measurements [6]. Hu, Duan and Zhou proposed a distributed asynchronous fusion algorithm by reconstructing the optimal centralized fusion result [7]. Beugnon, Singh, Llinas and Saha gave an adaptive track fusion algorithm [8], which is based on the requirements of the current system to select adaptively a track fusion algorithm. Li, Cheng, Zhang and Shen added the feedback structure on the basis of the adaptive track fusion algorithm [9]. Hao, Fawzi, Lefaudeux and Pollard proposed a new track fusion architecture using the split covariance intersection filter-information matrix filter [10]. Duraisamy, Schwarz and Wohler presented an overview of the track fusion algorithms to fuse two homogenous sensor systems [11]. Besides, scholars put forward many solutions to the track fusion problems under different environments. Chen and Bar-Shalom modified several track fusion algorithms accounting for the model mismatch among some of the local tracks [12]. Aziz proposed a new multiple decisions fusion rule in the distributed multiple sensor systems. The proposed fusion method derives the overall decision based on the multiple decisions from each individual sensor assuming that the probability distributions are not known [13]. Zhu, Zhai, Yu and Han addressed the estimation fusion when the cross-correlation of local estimation errors is partially known [14].Chen and Li focused on the tradeoff between the bandwidth and the tracking accuracy for the track fusion with communication constraints [15]. Watson, Rice and Alouani developed the solution for fusing multiple tracks from an arbitrary number of asynchronous measurements with a low complexity [16]. Chang, Chong and Mori focused on scalable fusion algorithms and conducted the analytical performance evaluation under different operating conditions [17]. Aeberhard, Schlichtharle, Kaempchen and Bertram presented a novel approach in a high-level sensor data fusion architecture for the automotive surround environment perception [18]. Quaranta and Balzarotti proposed an algorithm for the fusion of data originated by a bidimensional radar and infrared search [19]. Besides, the works in [20–26] contribute to the research of the information fusion problems.

Due to the complexity of targets and environments, track fusion requires multiple aspects of information. However, this does not mean that increasing the amount of information being fused always yields better results. In 1997, Hall and Llinas pointed out that the fusion of some sensor data may actually produce worse results than could be obtained by tasking the most appropriate sensor in sensor suite. This is caused by the attempt to combine the accurate data with inaccurate or biased data [27]. In dense sensor environments, there is a large amount of redundant information and the contribution of each sensor track to the system track is different. In some cases, some sensors at fault may increase the fusion time greatly, even lead to opposite results. However, most of the existing multi-sensor systems fuse all the available sensor tracks and treat all sensor data equally without regard of their different quality and different contribution to the system tracks. Actually, no matter what kinds of fusion technologies we adopt, the use of reliable information is the precondition of a successful fusion. If the quality of the data provided by the local sensors is different, the accuracy of system tracks will be different. In the dense sensor environments, the high-quality data is vital important to the validity of the fusion system. So a track fusion algorithm based on the reliability in dense sensor environments is proposed in this paper.

## Track Association

Throughout this paper, assume that *M* sensors observe *T* targets in clutter. The observation obtained in the discrete time is made up of several measurements, and some are from targets and the others from the clutter.

In the multi-sensor environments, each sensor detects all the targets in the surveillance region and forms the local tracks of these targets. The central node fuses the local tracks from the same target to acquire system tracks. So the track association should be considered at first to judge whether the local tracks from different sensors derive from the same target.

Assume the state estimation of a sample point from sensor *i* at time *k* is:

$$x_{{}_{i}}^{}(k)={\left(p_{1}(k),\;p_{2}(k),\;\cdots ,\;p_{n}(k)\right)}^{\text{T}}$$ (1)

where *p*_*m*_(1≤*m*≤*n*) is the feature of local tracks, and *n* is the number of features.

The corresponding distinguishing rate of sensor *i* is:

$$\Delta_{i}={\left(\delta_{1},\delta_{2},\cdots ,\delta_{n}\right)}^{\text{T}}$$ (2)

where *δ*_*m*_ is the corresponding distinguishing rate of each feature.

Define the statistical distance of two sample points:

d=ij2{‖xi(k)−xj(k)‖   i≠j‖Δi‖                       i=j (3)

Namely,

$$\left\{ \begin{array}{l} d_{ii}={\sqrt{\Delta_{i}^{\text{T}}\Delta }}_{i}\\ d_{ij}=\sqrt{{\left(x_{i}^{}(k)-x_{j}^{}(k)\right)}^{\text{T}}(x_{i}^{}(k)-x_{j}^{}(k))}\\ d_{ji}=\sqrt{{\left(x_{j}^{}(k)-x_{i}^{}(k)\right)}^{\text{T}}(x_{j}^{}(k)-x_{i}^{}(k))}\\ d_{jj}=\sqrt{\Delta_{j}^{\text{T}}\Delta_{j}} \end{array}\right.$$ (4)

If *d*_*ij*_≤min(*d*_*ii*_,*d*_*jj*_), the two sample points are associated, which means they are from the same target and should be fused.

## The State Estimation Fusion Based on the Reliability

### Outlier elimination

In order to improve the quality of local tracks, outliers are detected at first.

The expectation of the state estimation of target *t* at time *k* can be expressed as:

$${\bar{x}}^{t}(k)={\left({\bar{p}}_{1}(k),\;{\bar{p}}_{2}(k),\cdots ,\;{\bar{p}}_{n}(k)\right)}^{\text{T}}$$ (5)

where ${\bar{p}}_{i}(k)=\frac{1}{M}\sum_{i=1}^{M}p_{i}(k)$.

The average distance of the state estimation of target *t* at time *k* is:

$$S_{}^{t}(k)=\frac{1}{M}\sum_{i=1}^{M}\left\|x_{i}^{t}(k)-{\bar{x}}^{t}(k)\right\|$$ (6)

Compare $x_{i}^{t}(k)$ with ${\bar{x}}^{t}(k)$. If

$$\cdots \left\|x_{i}^{t}(k)-{\bar{x}}^{t}(k)\right\|<3S_{}^{t}(k)$$ (7)

then the value is reasonable. Otherwise, the sample point is regarded as an outlier and its value is replaced by ${\bar{x}}^{t}(k)$.

### Reliability calculation

In the multi-sensor track fusion systems, the trust level of the measurement results from the local sensors is called the reliability of the sensor. TFR constructs multi-sensor reliability judgment matrices for different targets, and acquires reliability of all local sensors for each target. If the interference is different, the reliability of the data provided by local sensors is different, and the accuracy of the system tracks will also be different, even if the local sensors have the same error. Based on this fact, TFR assumes that most of the local sensors are reliable. Then, the closer the measurement results from a local sensor is to the average value, the higher their reliability ratio is, and the larger the corresponding elements in the judgment matrix is. For the target, the sensor is more reliable.

The ratio of the state estimation distance of target *t* from sensor *i* and from sensor *j* at time *k* is defined as:

$$r_{ij}=\frac{\left\|x_{j}^{t}(k)-{\bar{x}}^{t}(k)\right\|}{\left\|x_{i}^{t}(k)-{\bar{x}}^{t}(k)\right\|}$$ (8)

For target *t*, the reliability judgment matrix of local sensors is:

$$R^{t}=\left[\begin{array}{cccc} 1 & r_{12} & \cdots & r_{1M}\\ r_{21} & 1 & \cdots & r_{2M}\\ \cdots & \cdots & \cdots & \cdots \\ r_{M1} & r_{M2} & \cdots & 1 \end{array}\right]$$ (9)

Normalize *R* ^*t*^, get ${\bar{R}}^{t}$, where

$${\bar{r}}_{ij}=\frac{r_{ij}}{\sum_{s=1}^{M}r_{sj}},\cdots i,j=1,2,\cdots ,M$$ (10)

Add the element of ${\bar{R}}^{t}$ by row, and get the transition vector $V^{t}=\left[\begin{array}{cccc} v_{1}^{t}, & v_{2}^{t}, & \cdots , & v_{M}^{t} \end{array}\right]{\;}^{\text{T}}$, where $v_{i}^{t}=\sum_{j=1}^{M}{\bar{r}}_{ij}$, *i*,*j* = 1,2,…,*M*. And $v_{{}^{i}}^{t}$ is the reliability of the state estimation of target *t* from sensor *i*.

Repeat this process, establish the reliability vector for each target, and get *T* reliability vectors, *V* ^*t*^(*t* = 1,2,…,*T*).

### State estimation fusion

Select [*M*/2] state estimations with high reliability in *V* ^*t*^(*t* = 1,2,…,*T*) for each target. Fuse the selected state estimations to acquire the system tracks, and abandon the rest state estimations with low reliability.

The global state estimation at *k* is:

$${\hat{x}}_{\text{TFR}}^{t}(k)=P_{j}^{t}(k){\left(P_{i}^{t}(k)+P_{j}^{t}(k)\right)}^{-1}x_{i}^{t}(k)+P_{i}^{t}(k){\left(P_{i}^{t}(k)+P_{j}^{t}(k)\right)}^{-1}x_{j}^{t}(k)$$ (11)

The global error covariance is:

$$P_{\text{TFR}}^{t}(k)=P_{i}^{t}(k){\left(P_{i}^{t}(k)+P_{j}^{t}(k)\right)}^{-1}P_{j}^{t}(k)$$ (12)

where $x_{i}^{t}(k)$ is the local state estimation and $P_{i}^{t}(k)$ is the local error covariance of target *t* from sensor *i* at time *k*.

## Experiments

### System description and initial settings

Let *x*(*k*) be the state vector at time *k*. The target model is expressed as:

x(k+1)=F(k)x(k)+G(k)u(k)+v(k) (13)

where *F*(*k*) is a state transition matrix, *G*(*k*) is an input control matrix, *u*(*k*) is a mobile acceleration input matrix, and *v*(*k*) is a discrete-time white noise sequence, which satisfies *E*(*v*(*k*)) = 0.

The measurement equation of sensor *i* is expressed as:

$$Z_{i}(k)=H(k)x(k)+w_{i}(k)$$ (14)

where *H*(*k*) is an observation matrix, and *w*_*i*_(*k*) is a Gauss observation noise with zero mean and variance *R*_*i*_(*k*). All the targets in the experiments are generated randomly according to the above target model.

In order to facilitate the problem discussion, assume all the state estimations sent into the fusion center are in the same coordinate system, all the sensors sample synchronously, and the delay time of data transmission is 0. The parameters of the sensors adopted in the experiments are exhibited in Table 1.

**Table 1:** The parameters of the sensors.

Sensor Number	Longitude(°)	Latitude(°)	Height(m)	Range error(m)	Azimuth error(°)	Pitch error(°)
1	122.1	40.5	0	120	0.6	0.6
2	122.4	41.5	0	180	0.9	0.9
3	122.7	41.9	0	100	0.5	0.5
4	123.0	42.9	0	200	1.0	1.0
5	123.3	43.3	0	140	0.7	0.7
6	123.6	44.3	0	160	0.8	0.8

### Experimental results and analysis

**Fig 1:**
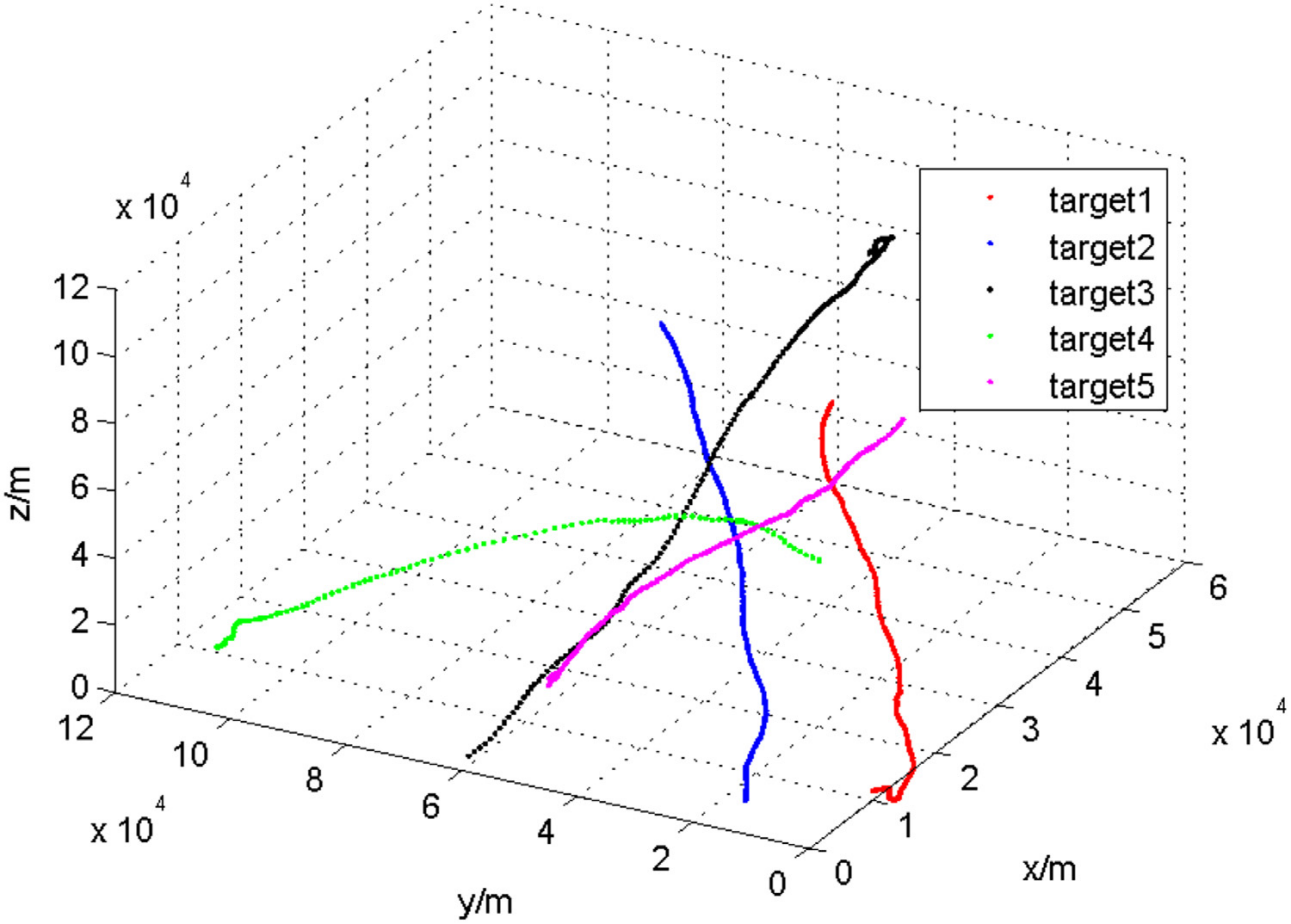
The target tracks with 5 targets.

**Fig 2:**
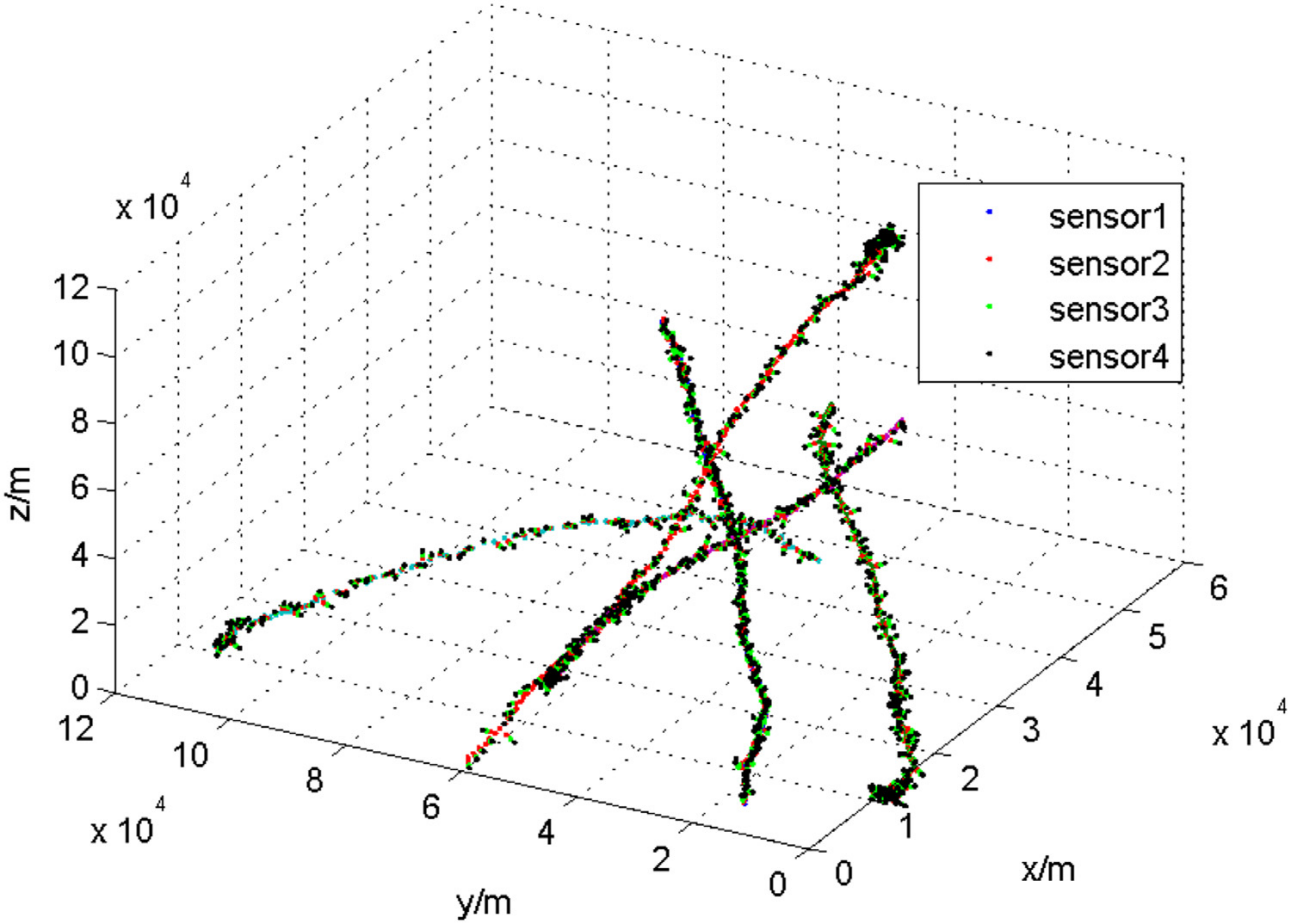
The observational data from local sensors.

The first experiment is designed to model four sensors to observe 5 targets at the same time, in which the first four sensors in Table 1 are adopted. The sampling interval is 1 second, and the target-tracking lasts 100 seconds. The targets run at a variable speed in the three-dimensional space. The target tracks are shown in Fig 1, and the observational data from local sensors are shown in Fig 2.

**Fig 3:**
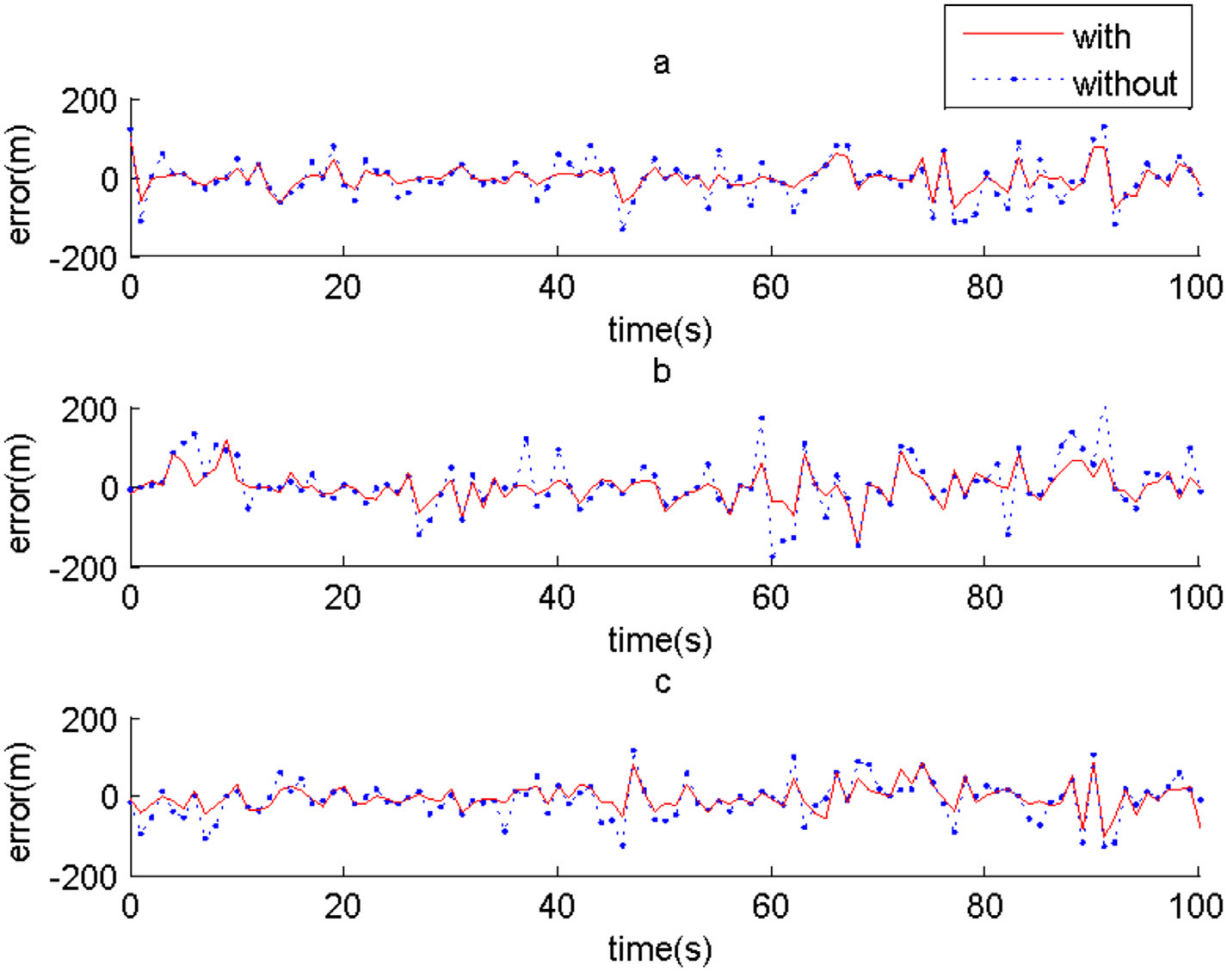
Comparisons of error between the cases with reliability analysis and without reliability analysis of target1. (a) In x-axis, (b) In y-axis, and (c) In z-axis.

In order to validate the performance, the algorithm is simulated 100 times by Monte Carlo method. Comparisons of error between the cases with reliability analysis and without reliability analysis in x-axis, y-axis and z-axis of target1 are respectively shown in Fig 3a, 3b and 3c. By the reliability analysis, the error of the system tracks is reduced greatly, which verifies that the reliability analysis can improve the accuracy of system tracks. For the other targets, there are similar conclusions. Namely, the reliability calculation of local tracks is a useful preliminary step to improve the accuracy of the track fusion.

**Fig 4:**
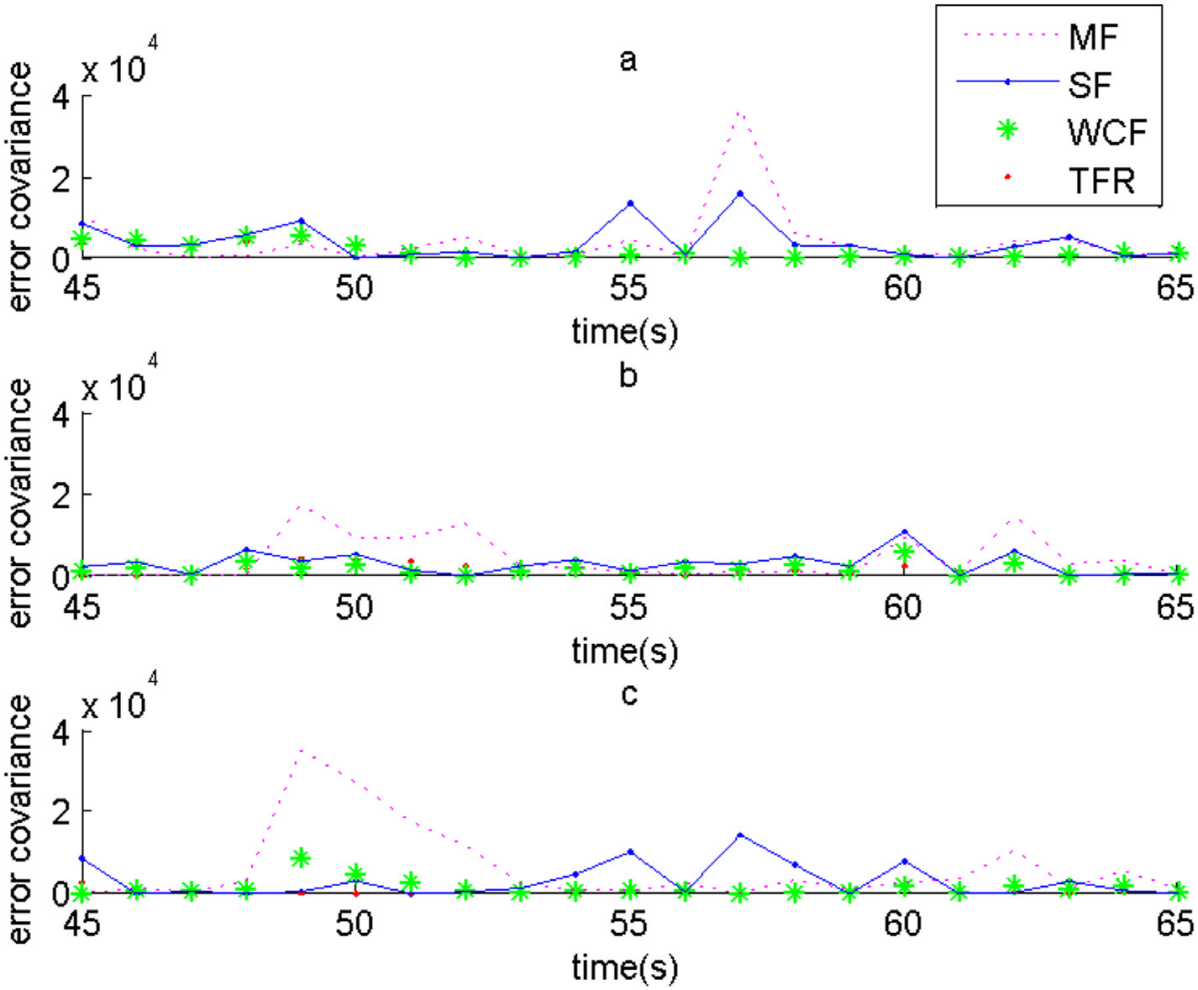
Comparisons of error covariance among MF, SF, WCF and TFR at the inflection points of target1. (a) In x-axis, (b) In y-axis, and (c) In z-axis.

Comparisons of error covariance among MF, SF, WCF and TFR at inflection points in x-axis, y-axis and z-axis of target1 are respectively exhibited in Fig 4a, 4b and 4c. We see TFR obtains the highest accuracy at the inflection points in general.

**Fig 5:**
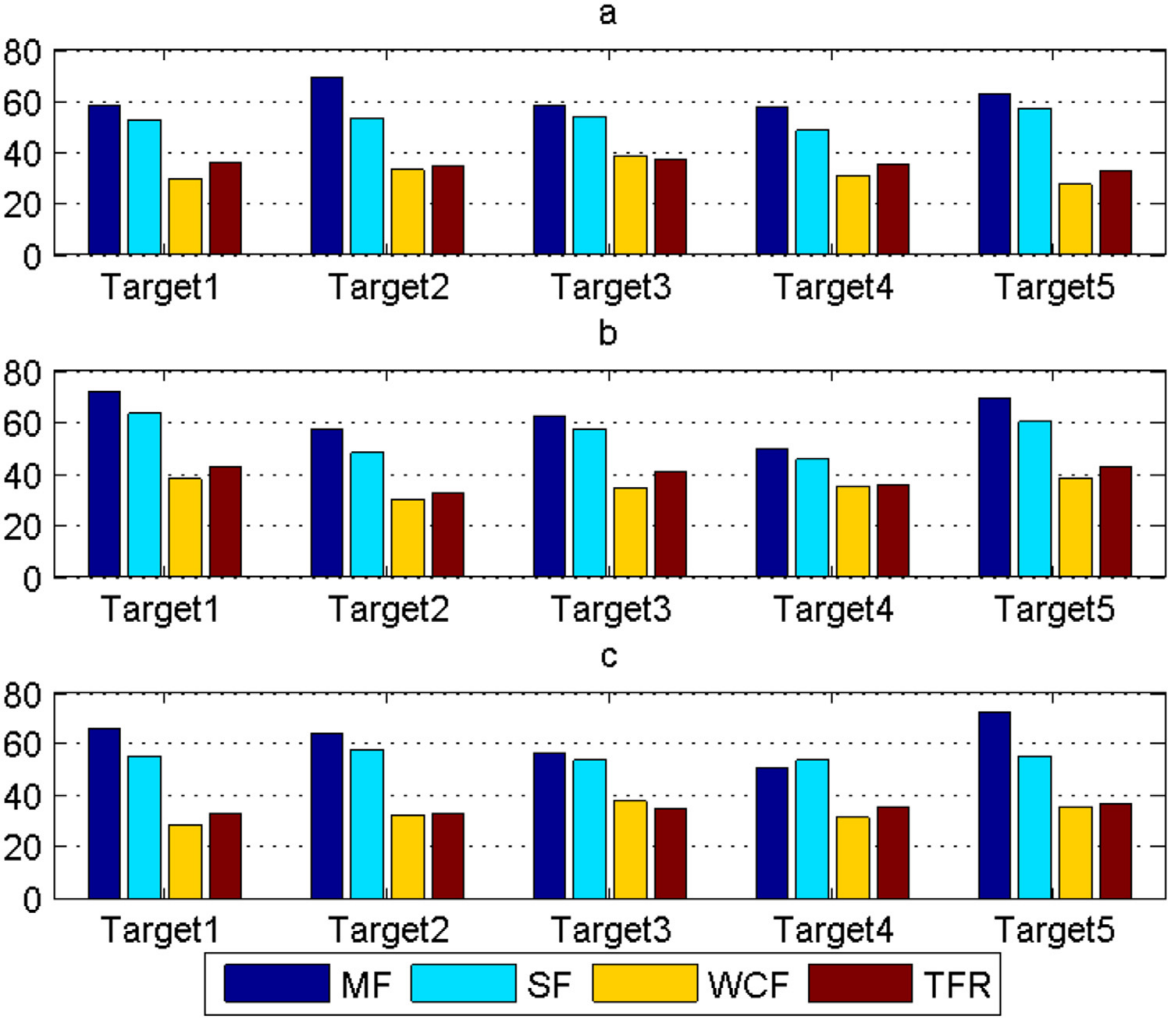
Comparisons of average error among MF, SF, WCF and TFR of 5 targets. (a) In *x*-axis, (b) In *y*-axis, and (c) In *z*-axis.

The average errors in *x*-axis, *y*-axis and *z*-axis of each target gained by MF, SF, WCF and TFR are exhibited in Table 2 and Fig 5. And Table 3 shows the average time for the fusion of all targets by MF, SF, WCF and TFR. From Table 2, Table 3 and Fig 5, we see that MF and SF have a fast speed, but a low accuracy. WCF possesses the highest accuracy, but with a relatively heavy computational cost. While, TFR balances the conflicts between the fusion accuracy and the computational burden.

**Table 2:** Comparisons of average error among MF, SF, WCF and TFR of 5 targets.

Algorithm	Target1			Target2			Target3			Target4			Target5		
	*x*	*y*	*z*	*x*	*y*	*z*	*x*	*y*	*z*	*x*	*y*	*z*	*x*	*y*	*z*
MF	58.29	71.47	65.85	68.75	56.86	63.78	58.29	62.19	56.11	57.46	49.28	50.70	62.38	69.21	72.11
SF	52.35	63.06	54.97	52.86	47.98	57.52	53.73	57.31	53.65	48.37	45.36	53.55	56.76	59.87	55.32
WCF	29.68	37.90	28.69	33.05	29.96	32.72	38.45	34.48	37.82	30.92	34.91	31.62	27.35	38.06	35.64
TFR	35.75	42.53	33.18	34.74	32.74	33.31	37.38	40.75	35.15	35.33	35.73	35.84	32.92	42.35	36.58

**Table 3:** Comparisons of fusion time among MF, SF, WCF and TFR of 5 targets.

Algorithm	Fusion time (s)
MF	46.61
SF	57.74
WCF	132.30
TFR	72.56

**Fig 6:**
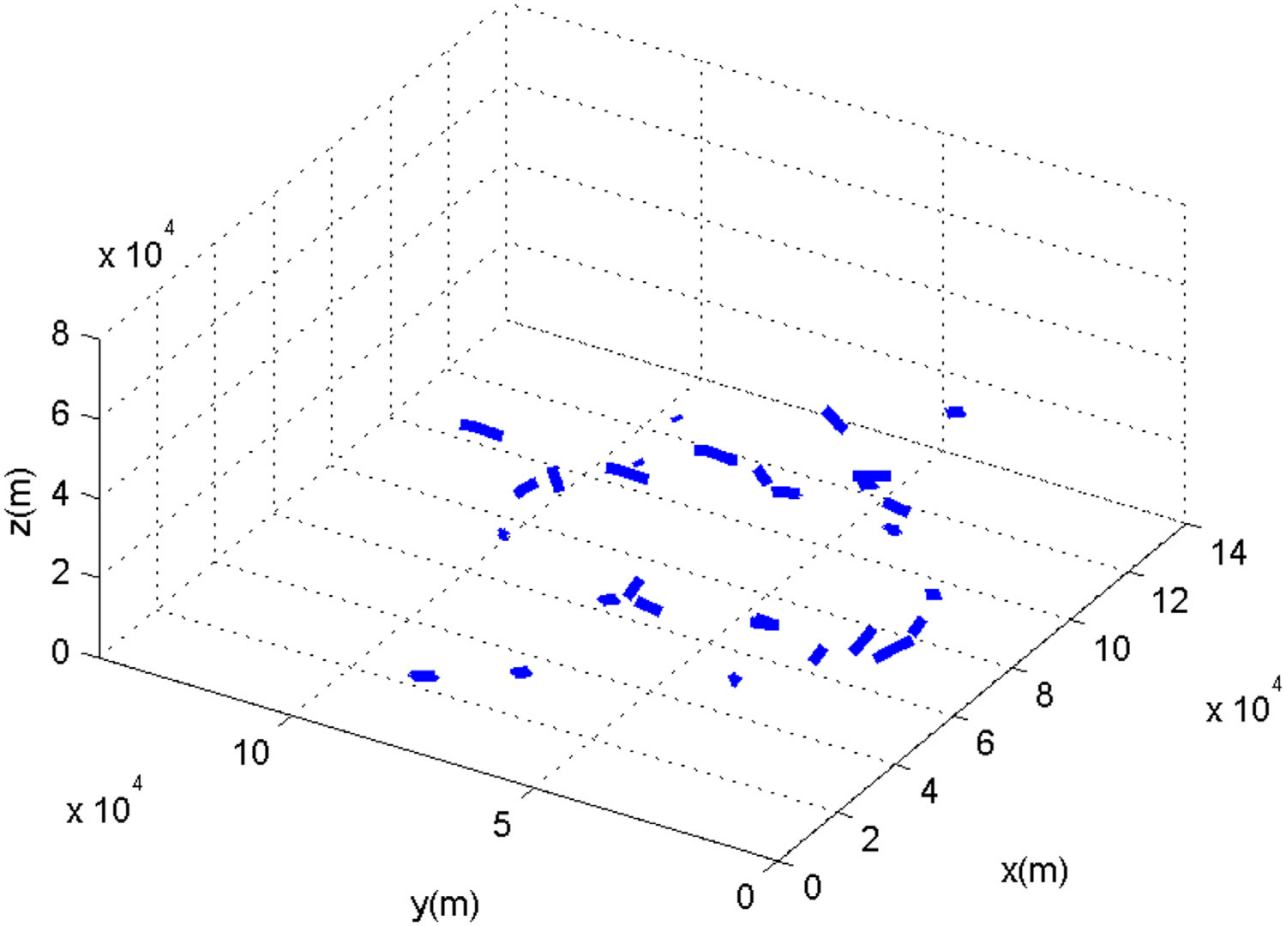
The target tracks with 30 targets.

Another experiment is done on larger datasets, in which 30 targets enter the surveillance space. The sampling interval is 1 second, and the target-tracking lasts 20 seconds. The targets run at a variable speed in the three-dimensional space. The tracks with 30 targets are shown in Fig 6.

Two cases are set in the experiment. Case1 is the dense sensor environment, in which six sensors observe the targets at the same time. The parameters of the six sensors are exhibited in Table 1. And Case2 is the sparse sensor environment, in which two sensors observe the targets at the same time. The first two sensors in Table 1 are adopted.

**Fig 7:**
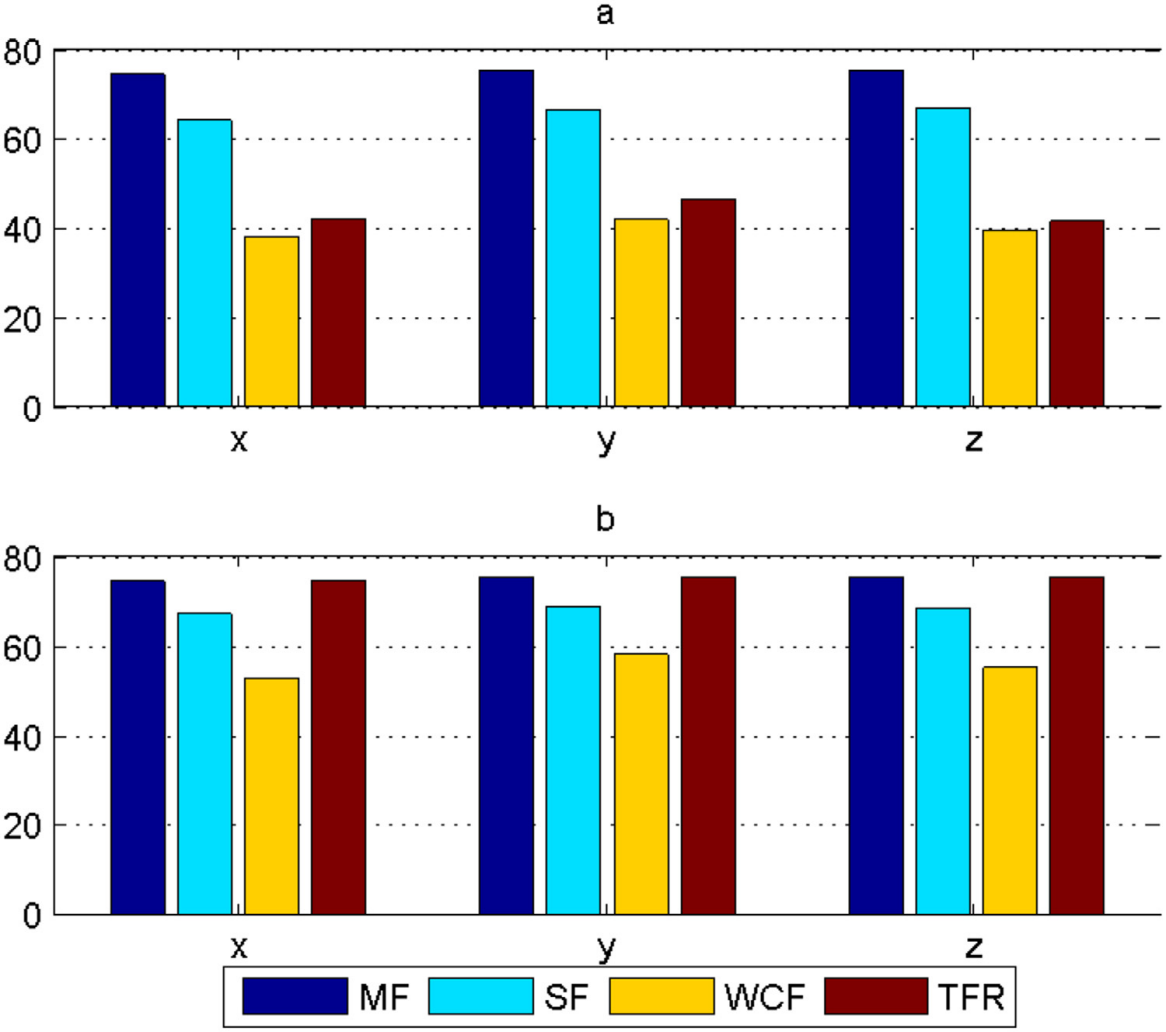
Comparisons of average error among MF, SF, WCF and TFR of 30 targets. (a) Case1, and (b) Case2.

In order to validate the performance, the algorithm is simulated 100 times by Monte Carlo method. Fig 7a and 7b show the comparisons of average error by 100 times simulation for fusing all targets by MF, SF, WCF and TFR in Case1 and Case2. Table 4 shows the comparisons of the fusion time by 100 times simulation for fusing all targets by MF, SF, WCF and TFR in Case1 and Case2.

**Table 4:** Comparisons of fusion time among MF, SF, WCF and TFR of 30 targets.

Algorithm	Case1(s)	Case2(s)
MF	107.49	26.83
SF	136.40	32.46
WCF	344.78	76.68
TFR	149.04	48.53

From the simulation results, we find that in the dense sensor environment, MF, SF and WCF all have the serious imbalance between the accuracy and the computational cost. Compared with them, TFR acquires a higher fusion accuracy with an acceptable computational cost. The success of TFR lies in that it excludes some low-reliability local tracks, which both reduces the fusion time and improves the fusion accuracy. In the sparse sensor environment with two sensors, TFR degenerates into MF, and costs more time. Thus, we draw a conclusion that TFR suits for dense sensor environments with a large amount of redundant information, and does not suit for sparse sensor environments with "poor" sensor information.

## Conclusion

At present, most track fusion algorithms take into account the adaptation and the completeness of the fusion strategies, with little thinking over the information quality provided by the local sensors. This paper differs from such approaches by introducing a track fusion algorithm based on the reliability from the view of improving the quality of local tracks. By abandoning the tracks with low reliability, the algorithm not only reduces the fusion complexity, but also facilitates the process of the fusion. The simulation results show the reliability analysis plays a significant role in improving the accuracy of system tracks, especially at inflection points, and TFR balances the conflicts between the accuracy and the computational cost in dense sensor environments.
